# Dataset of process-structure-property feature relationship for laser powder bed fusion additive manufactured Ti-6Al-4V material.

**DOI:** 10.1016/j.dib.2023.108911

**Published:** 2023-01-18

**Authors:** Qixiang Luo, Lu Yin, Timothy W. Simpson, Allison M. Beese

**Affiliations:** a Department of Material Science and Engineering, Pennsylvania State University, University Park, PA 16802, United States of America; b Department of Industrial and Manufacturing Engineering, Pennsylvania State University, University Park, PA 16802, United States of America; c Department of Mechanical Engineering, Pennsylvania State University, University Park, PA 16802, United States of America

**Keywords:** Additive manufacturing, Laser powder bed fusion, Processing parameters, X-ray computed tomography

## Abstract

The processing, structure, and property features for Ti-6Al-4V additively manufactured using laser powder bed fusion (L-PBF) over a range of processing parameter combinations are reported. In terms of processing, laser power and laser scanning speed were varied over a wide range, to investigate dense processing space as well as regimes likely to result in keyhole, lack of fusion, and beading up defects, which can occur during L-PBF. Archimedes measurements were used to measure porosity, while X-ray computed tomography (XCT) was used to quantify pore sizes, pore morphologies, and overall porosity, and finally, optical microscopy was used to quantify prior-β grain characteristics. Average pore size and shape, porosity, prior-β grain size and aspect ratio, and surface roughness for each processing parameter set are reported. Uniaxial tension tests and microhardness measurements were performed, with elastic modulus, yield strength, ultimate tensile strength, elongation to necking, elongation to fracture, and Vickers microhardness reported.

**Specifications Table**

**Table:** 

Subject	Manufacturing Engineering.
Specific subject area	Dataset with quantified processing, structure (microstructure, defect structure, surface roughness), and mechanical properties for laser powder bed fusion (L-PBF) Ti-6Al-4V [1,2].
Type of data	Table
How the data were acquired	Specimen fabrication: laser powder bed fusion metal additive manufacturing system (ProX DMP 320, 3D Systems, Inc.). Defect pore characterization: X-ray computed tomography (Phoenix v|tome|x L300 nano/micro-CT system, General Electric) with image processing software (Amira-Avizo 2020.2, Thermo Fisher Scientific [3]); Archimedes porosity measurements (OHAUS EX623). Grain characterization: optical microscope imaging (VHX-2000, Keyence) with imaging processing software (MIPAR v3.3.4 [4]). Mechanical testing: Vickers microhardness indentation (LECO LM 110AT); uniaxial tensile testing (MTS Criterion Model 45) with digital image correlation using a digital camera (Point gray GRAS-50S5M-C) with analysis software (VIC-2D/3D 6, Correlated Solutions).
Data format	Raw and analyzed
Description of data collection	Specimens were fabricated using a laser powder bed fusion AM system, using 42 different combination of processing parameters (laser power and scan speed). Internal pores were characterized using X-ray computed tomography and subsequent volume reconstruction of internal pores via image processing software. Metallurgical processing techniques (i.e., mounting, polishing, etching, optical microscope imaging, and image processing) were used to determine prior-β grain morphologies of the resulting material. Mechanical properties were measured using uniaxial tensile tests and microhardness indentation.
Data source location	Specimen fabrication: 3D Systems, 700 Marine Dr, Rock Hill, SC 29730, USA. CIMP-3D, 230 Innovation Boulevard, Penn State University, Innovation Park, PA USA. Defect pore characterization: Center for Quantitative Imaging (CQI), Pennsylvania State University, University Park, PA, USA. Grain structure characterization and mechanical testing: Multiscale Mechanics of Materials Lab, Pennsylvania State University, University Park, PA, USA.
Data accessibility	Repository name: Zenodo.org. Data identification number/DOI: 6587905 Direct URL to data: https://zenodo.org/record/6587905 Instructions for accessing these data: There are no specific restrictions or access controls for the data posted in the repository, all the data are available to access through the direct URL of the data repository, or search for the identification number in Zenodo platform.
Related research article	Luo, Q., Yin, L., Simpson, T. W., & Beese, A. M. (2022). Effect of processing parameters on pore structures, grain features, and mechanical properties in Ti-6Al-4 V by laser powder bed fusion. Additive Manufacturing, 102915. 10.1016/j.addma.2022.102915[1]

## Value of the Data

• The data provide quantitative information on the structure and mechanical properties that result from a range of processing conditions in laser powder bed fusion additive manufactured Ti-6Al-4V material, acquired through microstructural characterization using optical microscopy and X-ray computed tomography and mechanical testing.
• The data provided in this article provide insights on the process-structure-property relationship in additively manufactured Ti-6Al-4V over different processing ranges using laser powder bed fusion.
• The data can be used to support the selection and optimization of processing parameter in l-PBF of Ti-6Al-4V.
• The data can be used in the development or validation of data-driven models and integrated computational materials engineering (IMCE) software tools that connect processing, microstructure, and mechanical properties of l-PBF AM Ti-6Al-4V.

## 1 Objective

The objective for this dataset is to provide quantitative data on the microstructural features, including defect features, and mechanical properties, that result from a range of processing conditions in Ti-6Al-4V by laser powder bed fusion. These data can provide information to reveal the processing-structure-property relationships in this alloy produced via additive manufacturing.

## 2 Data Description

A total of 42 processing parameter sets (i.e., unique combinations of laser power and laser scan speed) was designed to probe a wide range of processing regimes (dense, lack of fusion porosity, keyhole porosity, beading up defects) were designed. Tensile specimens were fabricated with these processing parameter sets using a 3D System ProX DMP 320 laser powder bed fusion (L-PBF) additive manufacturing (AM) system. Both flat and round uniaxial tension specimens complying with ASTM E8/E8M [5] were fabricated, with each sample fabricated using one processing parameter set, followed by a 5-hour stress-relief heating at 670°C and 12-hour Argon atmosphere furnace cooling. The microstructures of these samples were characterized, and then samples were subjected to uniaxial tension. The resultant dataset consists of 7 processing condition features, data on 9 microstructural features, and data on 6 mechanical properties for each the processing set, as shown in Table 1. For Tables 2 to 9, numerical values for each of the process-structure-property (PSP) features is summarized in terms of both statistical mean and standard deviation. Indexing of these features, the corresponding PSP values, and the experimental/characterization methods are shown in Table 1.

**Table 1:** Processing, microstructural, and mechanical property features studied.

Feature type	Experimental devices	Extracted process-structure-property features	Index
Processing conditions	Prior fabrication designed processing parameters and descriptors.	Laser power P	Table 2
		Scan speed v	
		Hatch spacing h	
		Layer thickness l	
		Linear energy density LED (P/v)	
		Modified volumetric energy density MED (P/√v)	
		Laser power multiplied by scan speed Pv (Pv)	
Microstructural features	Archimedes testing	Pore volume fraction (Archimedes porosity)	Table 3
	X-ray computed tomography (XCT)	Pore volume fraction (XCT porosity)	Table 3
		Pore quantity, distribution over size ranges	Table 4
		Pore equivalent diameter	Table 5
		Pore sphericity	Table 5
		Pore projected area on the cross-section plane, orthogonal to the uniaxial loading direction	Table 5
		Surface roughness	Table 6
	Optical microscopy (OM)	Prior-β grain equivalent diameter	Table 7
		Prior-β grain aspect ratio	Table 7
Mechanical properties	Uniaxial tension tester (UT)	Yield strength	Table 8
		Ultimate tensile strength	Table 8
		Elongation to necking	Table 8
		Elongation to fracture	Table 8
		Elastic modulus	Table 9
	Hardness indentation	Vickers microhardness	Table 9

The data repository included the summary of processing set-dependent numerical mean and standard deviation values for each PSP features, as shown in Table 2 to 9, and the raw experimental data used to address the summarization. The higher-level overview of all PSP feature type, name and statistical values had been uploaded as the “Ti-6Al-4V PSP feature table” excel sheet in the repository. The raw experimental data were uploaded as sperate files, which included the original data from experimental devices, as shown in Table 1.

**Table 2:** Processing parameters and descriptors used for sample fabrication. Hatch spacing was 82 µm, and layer thickness 60 µm, for all samples.

	Processing parameters		Processing descriptors		
Parameter Set	Laser power P (W)	Scan speed V (mm/s)	LED (J/m)	MED ($J/\sqrt{m/s}$)	Pv (W*m/s)
1	275	800	344	307	220
2	275	760	362	315	209
3	275	720	382	324	198
4	275	680	404	333	187
5	275	640	430	344	176
6	275	600	458	355	165
7	275	840	327	300	231
8	275	880	313	293	242
9	275	920	299	287	253
10	275	960	286	281	264
11	275	1000	275	275	275
12	175	800	219	196	140
13	195	800	244	218	156
14	215	800	269	240	172
15	235	800	294	263	188
16	255	800	319	285	204
17	295	800	369	330	236
18	315	800	394	352	252
19	335	800	419	375	268
20	355	800	444	397	284
21	375	800	469	419	300
22	135	400	338	213	54
23	205	600	342	265	123
24	345	1000	345	345	345
25	415	1200	346	379	498
26	485	1400	346	410	679
27	500	1480	338	411	740
28	155	400	388	245	62
29	235	600	392	303	141
30	395	1000	395	395	395
31	475	1200	396	434	570
32	500	1290	388	440	645
33	115	400	288	182	46
34	175	600	292	226	105
35	295	1000	295	295	295
36	355	1200	296	324	426
37	475	1600	297	376	760
38	75	400	188	119	30
39	115	600	192	148	69
40	195	1000	195	195	195
41	235	1200	196	215	282
42	315	1600	197	249	504

**Table 3:** Porosity measured using X-ray computed tomography and the Archimedes method.

	XCT porosity (%)		Archimedes porosity (%)	
Parameter Set	Mean	Standard Deviation	Mean	Standard Deviation
1	0.8774	0.0452	1.3189	0.8816
2	1.9382	0.1495	1.8791	0.8775
3	2.3027	0.5489	2.1131	0.8748
4	1.6876	0.1638	2.3318	0.8728
5	2.0607	0.0928	2.5762	0.8700
6	1.7519	0.1885	2.7962	0.8677
7	1.2318	0.0008	1.2031	0.8836
8	1.1349	0.0489	1.0150	0.8853
9	0.5741	0.0222	0.8406	0.8877
10	0.5011	0.0508	0.7168	0.8880
11	0.2838	0.0003	0.6633	0.8878
12	0.7368	0.1135	1.0992	0.8835
13	0.5611	0.1192	1.1168	0.8839
14	0.4444	0.0017	0.9152	0.8853
15	0.7764	0.178	1.1020	0.8813
16	0.6089	0.0975	1.3991	0.8791
17	1.0879	0.3001	1.5610	0.8791
18	0.8338	0.0566	1.6791	0.8774
20	0.4546	0.0867	2.0801	0.8729
21	1.3623	0.5118	2.0075	0.8734
28	1.3591	0.2305	3.5888	0.8584
29	0.9718	0.2563	2.7060	0.8676
30	0.4503	0.0571	0.9296	0.8860
31	0.0523	0.009	0.6270	0.8883
32	0.0492	0.0083	0.5264	0.8901
33	1.5472	0.385	4.5078	0.8495
34	0.5205	0.1392	2.1473	0.8739
35	0.1538	0.0555	0.7814	0.8824
36	0.0653	0.0223	0.5854	0.8886
37	4.6949	0.0488	2.8603	0.8667
38	2.9905	0.1957	5.1688	0.8402
39	0.6683	0.1811	2.3110	0.8719
40	0.1141	0.016	0.3531	0.8910
41	0.0132	0.0056	0.3390	0.8917
42	0.0097	0.0038	0.3869	0.8909

**Table 4:** Total number of pores for each sample, and binned number of pores in four different equivalent diameter ranges.

	Pore quantity				
Parameter Set	All size range	*D*<200μm	*D*= [200,300) μm	*D*= [300,500) μm	*D*>500μm
1	44,006	44,006	0	0	0
2	57,383	57,383	0	0	0
3	57,874	57,874	0	0	0
4	67,526	67,526	0	0	0
5	93,003	93,003	0	0	0
6	62,355	62,355	0	0	0
7	56,069	56,069	0	0	0
8	50,236	50,236	0	0	0
9	35,273	35,273	0	0	0
10	22,806	22,806	0	0	0
11	17,670	17,670	0	0	0
12	42,124	42,122	2	0	0
13	41,642	41,641	1	0	0
14	31,408	31,408	0	0	0
15	48,679	48,679	0	0	0
16	29,954	29,954	0	0	0
17	40,214	40,214	0	0	0
18	44,417	44,417	0	0	0
20	28,498	28,498	0	0	0
21	38,997	38,997	0	0	0
28	68,407	68,405	1	0	1
29	53,425	53,425	0	0	0
30	22,826	22,826	0	0	0
31	2052	2052	0	0	0
32	1153	1153	0	0	0
33	76,806	76,788	6	8	4
34	40,141	40,140	0	1	0
35	16,145	16,145	0	0	0
36	7723	7723	0	0	0
37	11,801	10,864	691	210	36
38	3179	2847	180	117	35
39	46,274	46,158	91	25	0
40	11,094	11,094	0	0	0
41	2052	2052	0	0	0
42	288	288	0	0	0

**Table 5:** Defect pore size and morphology measurements. For the pore sphericity, both numerical and volumetric weighted mean values were analyzed, called mean and vol-mean here.

	Pore equivalent diameter (μm)		Pore sphericity			RMS value		
Parameter Set	Mean	Standard Deviation	Mean	Standard Deviation	Vol-mean	Mean $\left(\mu m^{2}\right)$	Maximum $\left(mm^{2}\right)$	Total $\left(mm^{2}\right)$
1	50	15	0.98	0.04	0.94	3798	0.76	280
2	52	16	0.97	0.05	0.92	3885	0.60	287
3	52	17	0.97	0.05	0.92	4046	1.55	339
4	52	18	0.97	0.05	0.92	4413	1.14	415
5	50	17	0.96	0.06	0.89	4040	1.72	534
6	53	19	0.97	0.05	0.91	4468	1.17	457
7	50	15	0.98	0.05	0.92	3448	0.48	208
8	49	14	0.98	0.05	0.91	3237	0.52	166
9	47	13	0.99	0.04	0.94	3240	0.40	129
10	47	12	0.99	0.03	0.96	2975	0.78	86
11	45	11	0.99	0.03	0.95	3616	1.29	85
12	47	12	0.99	0.03	0.96	3594	0.33	207
13	48	13	0.99	0.03	0.96	3507	0.67	209
14	47	13	0.99	0.03	0.96	3588	0.52	203
15	50	15	0.98	0.04	0.95	3731	0.63	244
16	48	13	0.99	0.03	0.95	3599	0.99	220
17	50	15	0.98	0.04	0.94	3900	0.85	286
18	50	16	0.98	0.04	0.94	4564	0.94	354
20	48	15	0.98	0.04	0.94	4447	1.18	333
21	50	16	0.98	0.04	0.93	4760	1.49	370
28	51	16	0.97	0.05	0.91	4311	0.80	502
29	50	16	0.98	0.05	0.93	3608	0.62	347
30	47	13	0.99	0.03	0.96	4074	0.69	116
31	47	17	0.99	0.03	0.94	6573	0.20	19
32	59	27	0.96	0.05	0.91	6129	0.20	9
33	45	13	0.99	0.04	0.79	4081	0.33	670
34	48	14	0.98	0.04	0.94	3903	0.43	373
35	45	11	0.99	0.02	0.97	3843	0.70	105
36	43	10	1	0.02	0.98	4126	0.20	42
37	105	69	0.85	0.12	0.60	21,643	1.96	339
38	97	101	0.81	0.2	0.29	7327	5.55	439
39	44	17	0.99	0.04	0.67	3315	0.24	339
40	44	11	1	0.02	0.97	3606	0.20	62
41	41	9	1	0.01	0.98	13,627	0.20	38
42	52	16	0.98	0.04	0.96	30,228	0.35	65

**Table 6:** Surface roughness measurements. Both arithmetic average (Ra) and root mean square average (RMS) values are given.

	Surface roughness (μm)			
	Ra value		RMS value	
Parameter Set	Mean	Standard Deviation	Mean	Standard Deviation
1	12.69	1.80	15.70	2.20
2	11.99	1.67	14.36	2.64
3	15.26	3.16	19.20	3.19
4	14.38	5.20	18.71	5.98
5	15.84	4.15	19.99	4.31
6	16.07	2.80	20.15	2.73
7	12.23	2.45	14.84	1.76
8	11.49	2.00	14.44	1.11
9	12.00	2.73	14.89	2.55
10	10.83	1.45	14.02	1.62
11	10.21	1.26	11.94	1.06
12	10.66	1.45	12.60	1.09
13	11.81	0.31	13.10	0.94
14	10.19	2.73	12.77	1.73
15	10.19	2.73	12.77	1.73
16	11.07	2.42	13.57	1.24
17	11.50	2.63	15.07	2.11
18	14.47	3.73	17.46	3.94
20	13.96	1.98	17.51	1.52
21	15.53	2.98	18.75	3.21
28	17.62	7.66	22.15	8.66
29	13.78	3.02	16.80	3.35
30	13.68	2.11	17.32	0.97
31	13.62	3.76	16.54	4.95
32	13.57	4.29	17.50	5.13
33	12.31	1.07	14.71	2.40
34	9.44	2.35	12.21	0.80
35	10.67	1.41	12.98	1.45
36	12.30	1.40	14.28	2.11
37	11.13	2.12	13.32	1.23
38	9.78	2.85	12.73	3.23
39	9.74	1.87	12.09	1.46
40	10.79	1.01	12.00	0.82
41	11.51	0.23	12.34	0.95
42	11.16	2.07	13.57	1.93

**Table 7:** Prior-β grain size and morphology.

	Grain equivalent diameter (μm)		Grain aspect ratio	
Parameter Set	Mean	Standard Deviation	Mean	Standard Deviation
1	204	114	1.92	0.66
2	205	105	1.98	0.74
3	188	88	1.88	0.60
4	167	63	1.89	0.58
5	168	64	1.89	0.58
6	173	63	1.91	0.61
7	171	64	1.89	0.60
8	164	62	1.88	0.60
9	185	71	1.88	0.57
10	202	105	2.05	0.74
11	251	122	1.95	0.63
12	240	128	2.10	0.79
13	222	95	1.99	0.64
14	189	67	1.96	0.68
15	208	102	2.06	0.74
16	197	95	1.96	0.69
17	229	101	1.93	0.63
18	168	55	1.95	0.62
19	179	69	1.96	0.63
20	172	67	1.91	0.61
21	224	108	1.88	0.61
22	264	115	2.46	1.02
23	177	58	1.93	0.62
24	188	74	1.88	0.58
25	211	102	1.89	0.57
26	257	129	1.95	0.60
27	196	98	1.88	0.57
28	182	75	1.91	0.59
29	191	73	1.89	0.61
30	164	58	1.90	0.59
31	207	92	1.91	0.60
32	176	77	1.86	0.57
33	210	116	1.92	0.62
34	165	63	1.89	0.57
35	223	98	1.93	0.64
36	209	91	1.90	0.58
37	224	129	1.89	0.57
38	207	96	2.24	0.94
39	231	110	1.97	0.68
40	205	101	1.87	0.57
41	228	103	1.93	0.61
42	234	113	2.00	0.67

**Table 8:** Measured yield and ultimate tensile strengths, and elongations to necking and failure.

	Yield strength (MPa)		Ultimate tensile strength (MPa)		Elongation to necking (%)		Elongation to fracture (%)	
Parameter Set	Mean	Standard Deviation	Mean	Standard Deviation	Mean	Standard Deviation	Mean	Standard Deviation
1	1021	6	942	10	6.15	0.08	10.70	0.76
2	1008	7	931	12	5.81	0.21	9.19	1.06
3	992	16	904	35	6.13	0.64	8.98	0.69
4	1009	20	929	21	5.52	0.17	8.50	1.33
5	995	27	914	35	5.37	0.38	7.23	1.22
6	982	14	892	44	5.27	0.32	6.95	0.54
7	1038	8	960	13	6.06	0.25	10.76	0.78
8	1053	9	974	19	6.13	0.16	11.87	1.03
9	1058	7	984	13	5.92	0.26	12.35	0.91
10	1070	10	994	14	6.37	0.47	14.03	0.62
11	1081	17	1000	19	6.33	0.46	13.91	0.84
12	1091	22	1025	18	5.86	0.42	12.39	2.05
13	1085	21	1014	19	6.45	0.19	12.53	2.20
14	1076	18	1005	15	6.47	0.22	12.82	0.96
15	1056	7	988	16	5.93	0.67	11.86	1.41
16	1041	15	965	18	6.13	0.28	10.71	1.33
17	1014	15	940	25	5.71	0.17	9.28	0.57
18	1001	7	927	17	6.02	0.32	9.70	0.85
19	1002	12	923	15	5.72	0.48	9.33	0.81
20	998	9	921	19	5.77	0.45	9.32	0.92
21	995	9	913	10	5.76	0.29	9.20	0.70
22	969	15	894	17	5.43	0.32	7.57	0.63
23	1015	8	936	8	5.69	0.22	8.03	0.81
24	1038	9	955	12	5.99	0.43	12.46	1.32
25	1054	21	977	20	5.82	0.19	13.58	1.54
26	1037	11	962	12	5.84	0.32	7.88	1.33
27	1015	24	946	25	4.21	1.07	4.47	1.18
28	965	8	897	7	4.12	1.39	5.10	2.19
29	1003	12	926	15	5.53	0.23	7.79	0.51
30	1036	8	955	13	6.24	0.31	12.39	1.09
31	1055	12	975	13	5.97	0.28	12.50	3.60
32	1033	15	959	18	6.00	0.36	10.81	2.73
33	954	30	901	20	3.30	2.36	4.38	3.78
34	1046	12	972	8	5.69	0.35	8.55	1.49
35	1074	19	993	22	6.07	0.28	13.46	1.52
36	1075	13	997	12	6.08	0.22	14.09	1.88
37	960	73	902	80	2.76	0.87	3.01	1.09
38	849	119	829	113	1.23	0.23	1.26	0.29
39	1040	33	989	33	2.71	0.56	3.05	0.78
40	1111	26	1047	23	6.29	0.42	14.22	1.40
41	1113	25	1048	24	6.23	0.41	15.38	1.30
42	1079	37	1014	29	5.56	1.63	11.77	5.47

**Table 9:** Measured Vickers microhardness and Young's modulus.

	Vickers microhardness (HV)		Young's modulus, E (GPa)	
Parameter Set	Mean	Standard Deviation	Mean	Standard Deviation
1	358	26	112	5
2	375	14	108	4
3	354	10	109	12
4	378	19	108	4
5	371	10	113	11
6	378	12	128	43
7	388	29	113	7
8	372	15	117	10
9	380	26	113	3
10	350	11	119	6
11	384	30	118	6
12	373	19	116	7
13	369	6	114	6
14	378	20	111	9
15	361	20	116	7
16	372	16	114	5
17	390	27	109	4
18	391	25	108	7
19	385	19	106	6
20	384	16	107	10
21	388	23	104	6
22	382	10	102	2
23	359	25	109	6
24	374	12	112	4
25	390	22	113	5
26	384	21	109	5
27	365	14	108	5
28	370	10	104	8
29	388	19	108	6
30	387	21	110	5
31	377	19	113	7
32	380	33	109	3
33	375	17	106	8
34	388	28	111	3
35	389	25	118	5
36	386	23	114	6
37	420	15	104	11
38	385	20	101	11
39	372	19	116	6
40	378	17	118	8
41	386	28	117	6
42	400	10	116	8

## 3 Experimental Design, Materials and Methods

### 3.1 Process map design

To identify different processing defect regimes for l-PBF AM Ti-6Al-4V [6], [7], [8], a total of 42 different processing parameter combinations (i.e., of laser power, and scanning speed) were designed. The hatch spacing, h, and thickness of each powder layer, l, were kept constant for all samples. Three different processing condition descriptors are utilized in this study: (1) linear energy density, (2) modified volumetric energy density, and (3) melt pool instability factor.

Linear energy density (LED), a metric to quantify the energy provided from the laser source to melt the layer below/substrate, is given as:

(1) $$\mathrm{LED}\;=\frac{P}{v}$$

LED assumes a constant penetration depth and layer thickness.

Modified volumetric energy density (MED) considers the laser energy distribution and variable penetration depth, and is given by [8].

(2) $$\mathrm{MED}\;=\frac{\beta }{h\sqrt{4\alpha \phi }}\cdot \frac{P}{\sqrt{v}}=C\left(\frac{P}{\sqrt{v}}\right)$$

where α and β are parameters related to the laser-powder interaction - laser absorptivity and the thermal diffusion of the powder - ϕ is the laser spot diameter, and C is a material constant.

Processing space boundaries based on LED and MED use the energy density to determine regimes for defects such as keyholing pores due to excessive energy densities, and lack of fusion due to insufficient energy densities resulting in incomplete fusion.

In addition to energy density-related defects, the beading-up or balling instability may also generate defects. This instability occurs when the melt pool elongates so much that it separates into multiple melt pools due to surface tension. The aspect ratio of the melt pool, defined as the length divided by width, is proportional to the laser power multiplied by scan speed (Pv), defined as [9], [10], [11]:

(3) $${\left(\frac{L}{w}\right)}^{2}=\frac{\epsilon e}{32\pi \kappa \alpha \Delta T}\cdot \left(\mathrm{Pv}\right)=C\left(\mathrm{Pv}\right)$$

where L and w are the length and width of the melt pool, respectively, ϵ is the laser absorptivity, e is Euler's number, κ and α are the conductivity and thermal diffusivity of the powder, ΔT is the temperature difference between melting point and room temperature, and C is a material constant.

### 3.2 Sample fabrication

Samples were fabricated by L-PBF AM system (ProX DMP 320 L-PBF system, 3D System, Inc., United States) and LaserForm®Ti Gr23(A) Ti-6Al-4V powder, with the elemental composition (wt.%) of 5.5–6.5 Al, 3.5–4.5 V, 0.25 Fe, 0.13 O, 0.08 C, 0.03 N, 0.012 H, balance Ti.

The contour processing parameters were held constant for all samples (275 W, 800 mm/s), while the processing parameters used to fabricate the sample interiors were varied among samples as shown in Table 2. A 245˚ hatch rotation was used between subsequent layers, with a constant layer height of 60 µm and constant hatch spacing of 82 µm.

Flat and round tensile samples complying with ASTM E8/E8M [5], were fabricated such that their tensile axes were aligned with the vertical build direction.

After fabrication, the as-built parts were subjected to a stress-relief heating at 670 °C for 5 h, followed by a 12-hour furnace cooling under Argon atmosphere. Samples were removed from the build plate using wire electrical discharge machining.

### 3.3 Microstructure characterization

Undeformed grip regions of fractured flat specimens were used for microstructural characterization. For each processing parameter set, one sample was used for metallographic investigation. The undeformed grip regions were first machined from the specimen after testing, and then mounted using an electro-hydraulic automatic mounting press (TechPress 3, Allied High Tech Products, Inc., United States) using a 1.25-inch mold assembly using black glass-filled epoxy powder. Standard metallurgical preparation grinding and polishing techniques were used with a final polishing using 0.05 µm colloidal silica suspension.

The polished samples were etched with Kroll's reagent (1 wt.% of hydrofluoric acid, 2 wt.% of nitric acid, and distilled water) for 30 s. Samples were imaged using a digital optical microscope (VHX-2000, Keyence, Inc., Japan). Optical images were analyzed via image analysis software [4] (MIPAR Image Analysis, MIPAR, Inc., United States), using a pre-processing contrast sharpen filter to identify the representative features, including prior-β grains and internal pores. The prior -β grain equivalent diameter and aspect was measured for all grains, with the mean and standard deviation for each processing parameter set output using a batch-processing automatic feature segmentation for all micrographs.

### 3.4 Internal pore characterization

Internal pores were characterized using X-ray computed tomography (XCT) (Phoenix v|tome|x L300 nano/micro-CT, General Electric, Inc., United States) with 170 kV and 55 mA. For each processing parameter set, one cylindrical geometry specimen was scanned with XCT. Each specimen was placed 35 mm away from the X-ray source and 700 mm away from the detector to yield a pixel pitch of 0.2 mm, which resulted in a detection voxel size of 10 μm. A 360˚ rotation of the specimen was applied to scan the specimen at gage region, using a 900 ms exposure time for each image, capturing a total of 900 images.

After the acquisition of image slices, three-dimensional reconstruction was achieved using the available software [12] (Phoenix datos|x 2.0 CT, Waygate Technologies, Inc., United States). Raw XCT data were analyzed through image processing software, including Amira-Avizo [3] (Amira-Avizo 2020.2, Thermo Fisher Scientific, Inc., United States), ImageJ [13] (ImageJ 1.8.0, National Institutes of Health, United States), and MATLAB [14] (MATLAB, MathWorks, Inc., United States).

Researchers have shown that pores or defects measuring 3 voxels or more in three orthogonal directions can be reliably detected with XCT [15]; thus, for the 10 μm voxel size used here, pores with all dimensions larger than 30 μm could be reliably detected. Quantitative pore features extracted include: (a) pore-level metrics: equivalent diameter, sphericity, and projected area onto the cross-section plane orthogonal to the tensile axis, and (b) specimen-level metrics: number of pores of different size ranges, and total porosity. Processing set-level statistical mean and standard deviation of pore-level metrics and specimen-level metrics were based on combining data from different instances of thresholding for image processing analysis. Archimedes porosity measurements, five tests per specimen, were also performed to characterize the internal porosity for each specimen, as a validation and comparison to XCT-measured porosity.

### 3.5 Mechanical testing

For each processing parameter set, two flat and two round specimens were tested under uniaxial tension. Prior to testing, each specimen was painted with white spray paint and a random black speckle pattern to provide features digital image correlation (DIC) could track during deformation. An electromechanical load frame (Criterion Model 45, MTS Systems, Inc., United States) with a 10 kN load frame was used to deform specimens under uniaxial tension at a strain rate of 3 × 10^−4^/s. For DIC, one (for flat specimens) or two (for cylindrical specimens) digital camera(s) (GRAS-50S5M-C, Point gray Research, Inc., British Columbia) were used to capture images of the specimen gage region during deformation at a rate of 1 Hz using (VIC-Gauge 2D/3D 6, Correlated Solutions, Inc., United States).

After testing, surface deformation fields were computed using analysis software (VIC-2D/3D 6, Correlated Solutions, Inc., United States). For all samples, a vertical 24 mm extensometer was used to measure the axial strain with a cubic B spline interpolation algorithm. A subset size of 29 pixels and a step size of 7 pixels were used to reach pixel resolution ranged from 0.0157 to 0.0259 mm/pixel. The stress-strain response for each specimen was constructed using exported data from the test, and yield strength, ultimate tensile strength, elongation at necking, elongation to failure, and elastic modulus were extracted. Processing set-level statistical mean and standard deviation of these mechanical properties were summarized based on four tests per processing condition. Microhardness measurements (LM 110AT, LECO, Inc., United States) were taken for each processing parameter set after polishing. For each sample, 10 indentations at different sample locations were made using a 100 gf load applied for 10 s load time.

## Ethics Statements

No human subjects, animal experiments and data collected from social media platforms were involved in this work.

## CRediT authorship contribution statement

**Qixiang Luo:** Writing – original draft, Visualization, Validation, Methodology, Investigation, Formal analysis, Data curation. **Lu Yin:** Investigation, Formal analysis. **Timothy W. Simpson:** Writing – review & editing, Funding acquisition, Conceptualization. **Allison M. Beese:** Writing – review & editing, Supervision, Resources, Project administration, Methodology, Funding acquisition, Conceptualization.

## Declaration of Competing Interest

The authors declare that they have no known competing financial interests or personal relationships that could have appeared to influence the work reported in this paper.

## Data Availability

Data on the effect of processing parameters on pore structures, grain features, and mechanical properties in Ti-6Al-4V by laser powder bed fusion (Original data) (Zenodo).
